# Supplementary material for: COVID-19 Infection in Pregnancy: PCR Cycle Thresholds, Placental Pathology, and Perinatal Outcomes
Source: Viruses. 2021 Sep 21;13(9):1884. doi: 10.3390/v13091884 (PMC8473449; doi:10.3390/v13091884)
Supplement: Supplementary file 1 [file viruses-13-01884-s001.zip › viruses-1354008-supplementary.pdf]

Supplementary Materials:

Table S1. Symptomatology.

Symptoms	COVID-19 Positive (299) Mean ± SD N (%)	COVID-19 Negative (828) Mean ± SD N (%)	All Patients (1127) Mean ± SD N (%)	*p*
Cough	7 (2.4)	1 (<1)	8 (<1)	0.001
Fever	9 (3)	7 (0.9)	16 (1.4)	0.018
Headache	13 (4.4)	15 (1.8)	28 (2.5)	0.019
Dyspnea	4 (1.3)	0	4 (<1)	0.005
Myalgias	3 (1)	0	3 (<1)	0.019
Rhinorrhea	1 (<1)	0	1 (<1)	0.266
Diarrhea	2 (<1)	1 (<1)	3 (<1)	0.174
Thrombosis	1 (<1)	1 (<1)	2 (<1)	0.466
Neurological symptoms	2 (<1)	3 (<1)	4 (<1)	0.613
Arrhythmia	2 (<1)	2 (<1)	4 (<1)	0.578
Lethargy	0	0	0	omitted

Table S2. Stepwise logistic regression for COVID19 manifestations and PCR positivity.

Parameter		Estimate	Standard Error	Wald Chi-Square	*p*
Intercept		15.9076	477.3	0.0011	0.9734
Cough	Yes	2.845	1.082	6.9	0.0085

Table S3. ANOVA.

	F	*p*	Partial Eta Squared
Pre-pregnancy BMI	0.057	0.811	0.001
Asymptomatic vs. asymptomatic	0.581	0.448	0.006

Table S4. Logistic regression model for preeclampsia and preterm birth.

Parameter	Estimate	SE	Wald Chi-Square	*p*	OR	Lower Bound CI 95%	Upper Bound CI 95%
Preeclampsia							
Symptomatic for COVID-19	2.7	0.93	8.44	0.004	14.72	2.40	90.4
Preterm delivery							
Preeclampsia	3.18	1.43	4.9	0.027	24	1.44	399
Gestational age at triage	−0.66	0.22	11.04	0.001	0.514	0.34	0.76
